# Natural orifice specimen extraction for diverticular disease: technique, outcomes and role of inflammatory markers

**DOI:** 10.1007/s00464-025-11803-4

**Published:** 2025-05-20

**Authors:** Mina Sarofim, Jasmine Mui, John Cartmill, Andrew Gilmore

**Affiliations:** 1 Department of Colorectal Surgery, Liverpool Hospital, Sydney, Australia; 2 School of Medicine, University of New South Wales, Sydney, Australia; 3 School of Medicine, University of Sydney, Sydney, Australia; 4 School of Medicine, Macquarie University, Sydney, Australia; 5 Department of Colorectal Surgery, Macquarie University Hospital, Sydney, Australia

**Keywords:** Natural orifice specimen extraction, Nose, Diverticular disease, Minimally invasive, Colectomy, Inflammatory response

## Abstract

**Background:**

Minimally invasive colectomy is common for diverticular disease. Natural orifice specimen extraction (NOSE) is an innovative adjunct that avoids the morbidity of abdominal incisions. The aim of this study is to evaluate the outcome of NOSE in laparoscopic surgery for complications of diverticular disease, and assess the role of post-operative inflammatory markers.

**Methods:**

A multi-centre prospective study was conducted from 2012 to 2024. Consecutive patients who underwent emergency and elective NOSE colectomy for diverticular disease were included. Demographics, surgical techniques, post-operative complications and biochemical results were analysed.

**Results:**

NOSE colectomy was successful in 99.4% of patients (171/172), with a mean age of 59.9 years. Indications for surgery were phlegmon (35%), recurrent diverticulitis (27%), stricture (21%), fistulae (14%) and haemorrhage (2%). Mean length of stay was 5.7 days (SD 3.8), and anastomotic leak rate was 1.8%. Specimen longitudinal splitting increased operative time (254 vs. 220 min, *p* < 0.01) and length of stay (6.6 vs. 5.3 days, *p* = 0.02). Significantly higher inflammatory markers were observed in the longitudinal split group on post-operative days 2–4 without increased complication or anastomotic leak rates.

**Conclusion:**

NOSE colectomy demonstrates excellent perioperative outcomes in this large series and is an effective approach for diverticular disease. Specimen debulking facilitates successful NOSE procedures, with expected increases in inflammatory markers which are not associated with higher complication rates.

Diverticulosis of the colon is remarkably common due to outpouching of the mucosa through openings in the muscular layer of the bowel. In addition to genetic predisposition, in Western countries, it is significantly associated with increasing age, obesity, and environmental factors such as diet and lack of exercise [1]. A proportion of these will develop symptomatic disease as a sequela of their diverticuli, most commonly diverticulitis or phlegmon/abscess formation (5–10%), bleeding (5%) or fistulae and strictures (4%) [1–3]. There is an evidence-based consensus on the role of surgery in diverticular disease; immediate resection should be considered in haemodynamically unstable or septic patients either by open or laparoscopic approach, and with or without primary anastomosis. Electively, surgical resection is considered on an individualised basis for symptomatic patients with radiological or endoscopic signs of ongoing inflammation, persistent abscesses, strictures or fistulae, best performed by minimally invasive approach if feasible [3].

Natural orifice specimen extraction (NOSE) is an innovative adjunct to laparoscopic or robotic colectomy in both emergency and elective surgery. During minimally invasive surgery, resected segments of bowel and the associated mesentery are traditionally removed via extraction incision through the abdominal wall, the length of which is dependent on the diameter of the specimen. Regardless of the orientation or location of these incisions, they are associated with post-operative pain, risk of surgical site infection or dehiscence and long-term risk of incisional hernia [4–6]. NOSE challenges this paradigm by utilising the orifice of an existing hollow viscus, such as the anus or vagina, as the pathway for specimen removal to avoid the associated morbidity of extraction incisions [7, 8].

There is an emerging body of literature which demonstrates NOSE feasibility for both benign and neoplastic colectomy, typically for patients with small lesions or specimens. Avoiding large extraction incisions to extract bulky specimens in patients with complications of diverticular disease has its advantages, but one must also acknowledge the potential challenges: steep learning curve, technical difficulty, obligatory intracorporeal anastomosis and the management of bulky specimens which are often phlegmonous and indurated from recurrent infection [8–11]. There is a paucity of data describing the morbidity, post-operative results and systemic inflammatory response in this cohort. The aims of this study are to evaluate the outcome of NOSE in laparoscopic surgery for complications of diverticular disease, describe advanced surgical techniques to facilitate specimen extraction and quantify the significance of post-operative inflammatory markers.

## Methods

### Study design

A prospective cohort study was performed across four tertiary colorectal surgery units in New South Wales, Australia between January 2012 and January 2024. The study was reported in compliance with preferred reporting of case series in surgery (PROCESS) guidelines [12], and study was preregistered in Open Science Framework no. p678f.

### Participants

All consecutive patients who underwent laparoscopic NOSE colectomy were eligible. Inclusion criteria are as follows: adults (age > 18), minimally invasive NOSE colectomy performed for complications of diverticular disease (phlegmon, bleeding, fistulae, stricture), planned elective surgery or emergency. Patients with suspected or confirmed left-sided malignancy were excluded.

The study obtained ethical approval from the Local Health Districts (Research Ethics and Governance Information System no. ETH01015), and informed consent was obtained preoperatively from each patient. A priori protocol followed local hospital guidelines.

### Variables

Collected data included patient demographics, intraoperative surgical approach and stapling technique, histopathology, biochemical results, including white cell count (WCC) and C reactive protein (CRP), and post-operative length of stay. Perioperative complications were defined as Clavien-Dindo Classification [13] Grade III (requiring surgical, endoscopic or radiological intervention) or Grade IV (requiring intensive care or organ support). Anastomotic leaks encompassed either clinical or radiological leaks.

### Outcomes

The primary endpoints of the study were post-operative length of stay, morbidity rate and biochemical inflammatory response. The secondary endpoints were outcomes of stapling technique and requirement for advanced debulking methods. The cohort was also divided into two subgroups based on whether the colon specimen was longitudinally split during extraction (Group A) versus those that were not (Group B). This was determined by the primary surgeon intraoperatively.

### Statistical methods

Statistical analysis was done with SPSS version 28 (IBM, USA). For comparison of binary variables, Chi square test was used. Continuous data were tested for normal distribution; if present, t test and ANOVA were used to compare groups, and mean and standard deviation [SD] are reported. If data were not normally distributed, Mann–Whitney and Kruskal–Wallis test were used, and median and range [R] are reported. The significance value was *p* < 0.05.

### Operative technique

All patients received pre-operative mechanical bowel preparation without oral antibiotics. Prophylactic intravenous antibiotics are given as per local guidelines and patients are positioned in the modified Lloyd-Davies position on a vacuum bean bag under general anaesthesia. On-table CO^2^ colonoscopy to the caecum is performed to exclude neoplastic disease, even if recently performed. If colonoscopy is not possible pre-laparoscopy, it is performed mid-operation after mobilisation of the sigmoid colon and prior to vascular transection. Dilute iodine lavage is used to decontaminate the colon during scope withdrawal. 5 mm ports are inserted according to surgeon preference, and colon mobilisation is performed in conventional fashion (including full mobilisation of the splenic flexure). Depending on the length of diverticular disease, the intended proximal resection margin is approximated to the intended rectal transection to ensure sufficient mobilisation for a tension-free anastomosis. The peritoneal reflection defined height of rectal resection, where high anterior was above and low anterior below. The mesocolon is next divided with meticulous attention to vascular demarcation. In contrast to malignant pathology, the inferior mesenteric artery and vein are preserved to ensure optimal perfusion and preservation of hypogastric nerves [11].

Nylon tape is placed to occlude the proximal and distal ends of the specimen. A Harmonic™ scalpel (Ethicon) is used to divide the proximal colon, and a 3.0 PDS Stratafix™ purse-string suture is placed laparoscopically. The rectum is similarly divided with Harmonic™ approximately 20 mm proximal to the intended area of anastomosis. Meticulous suction manages luminal spillage.

**Fig. 1:**
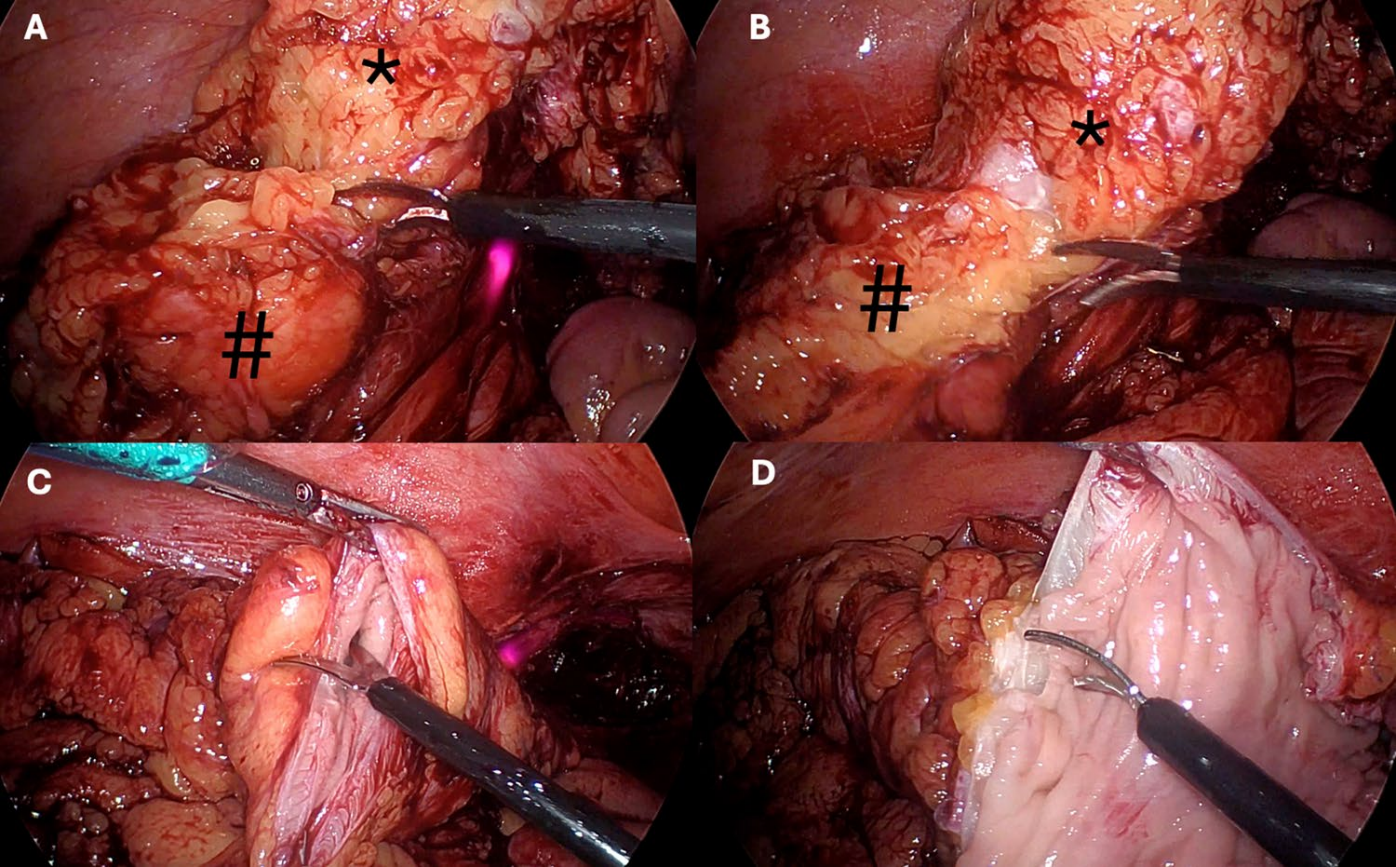
Laparoscopic view of advanced debulking techniques. (* colon, # mesentery). **A** Commencing mesocolic defatting with scissors. **B** Continuing mesocolic defatting proximally. **C** Commencing longitudinal splitting with scissors. **D** Continuing longitudinal splitting proximally

In situations where intact NOSE is not possible due to large specimen size (such as phlegmon), additional manoeuvres are used prior to intestinal transection. These manoeuvres are aided by three-point fixation; the surgeons left hand proximally, the rectum distally and the assistant’s grasper medially on colon, close to the dissection position. Mesocolic defatting (Fig. 1A, B) is performed initially with laparoscopic scissors, from inferiorly to superiorly. Usually, a tail of mesocolon is left attached just below the proximal transection point, as it can usually follow the specimen out during NOSE. Longitudinal splitting (Fig. 1C, D) of the colon is subsequently considered if the intestinal specimen remains too large for NOSE. Again employing three-point fixation, the colon is cut longitudinally into two (or rarely three) long pieces to allow safe retrieval (nylon tape is not required in this instance). Fastidious suction is required for mucous spillage. Diverticular faecoliths are carefully checked for, counted and removed later.

An XS (extra small) Alexis® Wound Protector (Applied Medical Resources Corporation) is modified by adding a No.5 Ethibond suture opposite the silk tether on the green ring. This allows deformation of the green ring into an ellipse for atraumatic insertion and removal anally via the rectal stump. Rampley forceps are passed through the Alexis® to grasp and retrieve the specimen(s). A circular anvil is passed into the pelvis using the Rampley and placed within the proximal colon. The purse-string suture is tied and anvil security reinforced with 0 PDS Endoloop™ ligature (Ethicon). The Alexis® is collapsed to remove, using the Rampley on the Ethibond tie with counter traction on the silk tether. A video demonstration of the key steps of this technique has been published by our group previously [14].

Typically, a double staple colorectal anastomosis is performed, whereby the rectal stump is closed with an endoscopic linear stapler (passed via a temporary oblique 12 mm port from the right upper quadrant), and a circular end-to-end stapler is utilised. A single staple technique is reserved for difficult rectal stumps, which replaces the endoscopic linear stapler with purse-string closure of the rectum. If a triple staple technique is required, a proximal staple line is made after anvil insertion via colotomy, to construct a side-to-end anastomosis. A flexible sigmoidoscopy is used to perform an air leak test and assess mucosal blood supply or bleeding. Post-operative care includes enhanced recovery after surgery (ERAS) principles, and laboratory investigations are guided by individual patients’ clinical progress, with diet upgrade as tolerated. Intravenous antibiotics are continued for 48–72 h depending on the degree of contamination.

## Results

### Participants

172 consecutive patients had an attempted laparoscopic NOSE procedure for complications of diverticular disease during the study period, and only one was converted to an abdominal extraction early in the series. 171 patients (99.4%) were therefore included in the analysis; mean age was 59.9 years, and 100 (58%) were female. Patient demographics, primary indication for surgery and additional complications of diverticular disease identified during surgery are listed in Table 1. Histopathology confirmed no cases of malignancy.

**Table 1:** Patient demographics of the entire cohort

Characteristic	Cohort (*n* = 171)
Mean age (years, [SD])	59.9 [12.5]
Females (*n*, [%])	100 [58]
Median BMI (kg/m^2^ [R])	29.5 [21.0–47.8]
Elective procedure (*n* [%])	157 [92]
Primary indication for surgery (*n* [%])	
Diverticular phlegmon	60 [35]
Recurrent diverticulitis	47 [27]
Stricture	36 [21]
Colo-vesical fistula	20 [12]
Colo-vaginal fistula	3 [2]
Recurrent Haemorrhage	3 [2]
Other	2 [1]
Additional diverticular complications (*n* [%])	
Stricture	19 [11]
Diverticular phlegmon	13 [8]
Abscess	4 [2]
Colo-enteric/colo-colonic fistula	3 [2]
Diverticulitis	2 [1]
Colo-vesical fistula	1 [1]

*SD* standard deviation, *n* number, *BMI* body mass index, *R* range

### Descriptive data

Characteristics of the surgical procedures are summarised in Table 2. Across the entire cohort, the mean operating time was 232 min. High anterior resection in 120 patients (70%) and low anterior resection in 49 (29%). Two patients required a total colectomy and ileorectal anastomosis, and two patients required both low anterior resection and right hemicolectomy. Two of the patients who had a hysterectomy had trans-vaginal NOSE, and the remainder of the cohort had transrectal. Anastomosis technique was single staple in 29 (17%), double staple in 140 (82%) and triple staple in 2 (1%). The colectomy specimen was retrieved without debulking manoeuvres in only 39 patients (23%). Longitudinal splitting of the specimen was performed independently in 7 cases (4%) and combined with mesocolic defatting in another 50 cases (29%). One patient required diverting ileostomy.

**Table 2:** Description of surgical procedures for entire cohort

Operative factor	Cohort
Mean operating time (mins [SD])	232 [59]
Ureteric catheter insertion (*n* [%])	63 [37]
Median ports (*n* [R])	4 [4–6]
Resections (*n* [%])	
High anterior	120 [70]
Low anterior	49 [29]
Total colectomy	2 [1]
Anastomosis staple technique (*n* [%])	
Single	29 [17]
Double	140 [82]
Triple	2 [1]
Specimen debulking (*n* [%])	
Group A (specimen split)	
Longitudinally split alone	7 [4]
Defatting and splitting	50 [29]
Group B (non-split)	
Mesocolic defatting	75 [44]
No debulking required	39 [23]
Additional procedures (*n* [%])	
Adhesiolysis > 2h	8 [5]
Hysterectomy/Oophorectomy	5 [3]
Cholecystectomy	3 [2]
Right hemicolectomy	2 [1]
Appendicectomy	2 [1]

*mins* minutes, *n* number

### Outcome data

The mean length of hospital stay was 5.7 days across the cohort (Table 3). There were no 30-day mortalities, and 11 patients (6.4%) experienced a Clavien-Dindo grade III–IV complication which included 3 anastomotic leaks (1.8%). Highest mean WCC occurred on day one (12.4 × 10^9^/L), whereas mean CRP peaked on day two (171 mg/L).

**Table 3:** Post-operative outcomes of the cohort

Outcome	Cohort
Mean length of stay (days [SD])	5.7 [3.8]
Complications ~ (*n* [%])	11 [6.4]
Anastomotic leak (*n* [%])	3 [1.8]
Ureteric injury (*n* [%])	1 [0.6]
Small bowel obstruction (*n* [%])	4 [2.3]
Anastomotic bleed (*n* [%])	2 [1.2]
Venous thromboembolism (*n* [%])	1 [0.6]
30 day mortality (*n* [%])	0 [0]

*SD* standard deviation, *n* number, ~ Clavien-Dindo III–IV

### Subgroup analyses

**Fig. 2:**
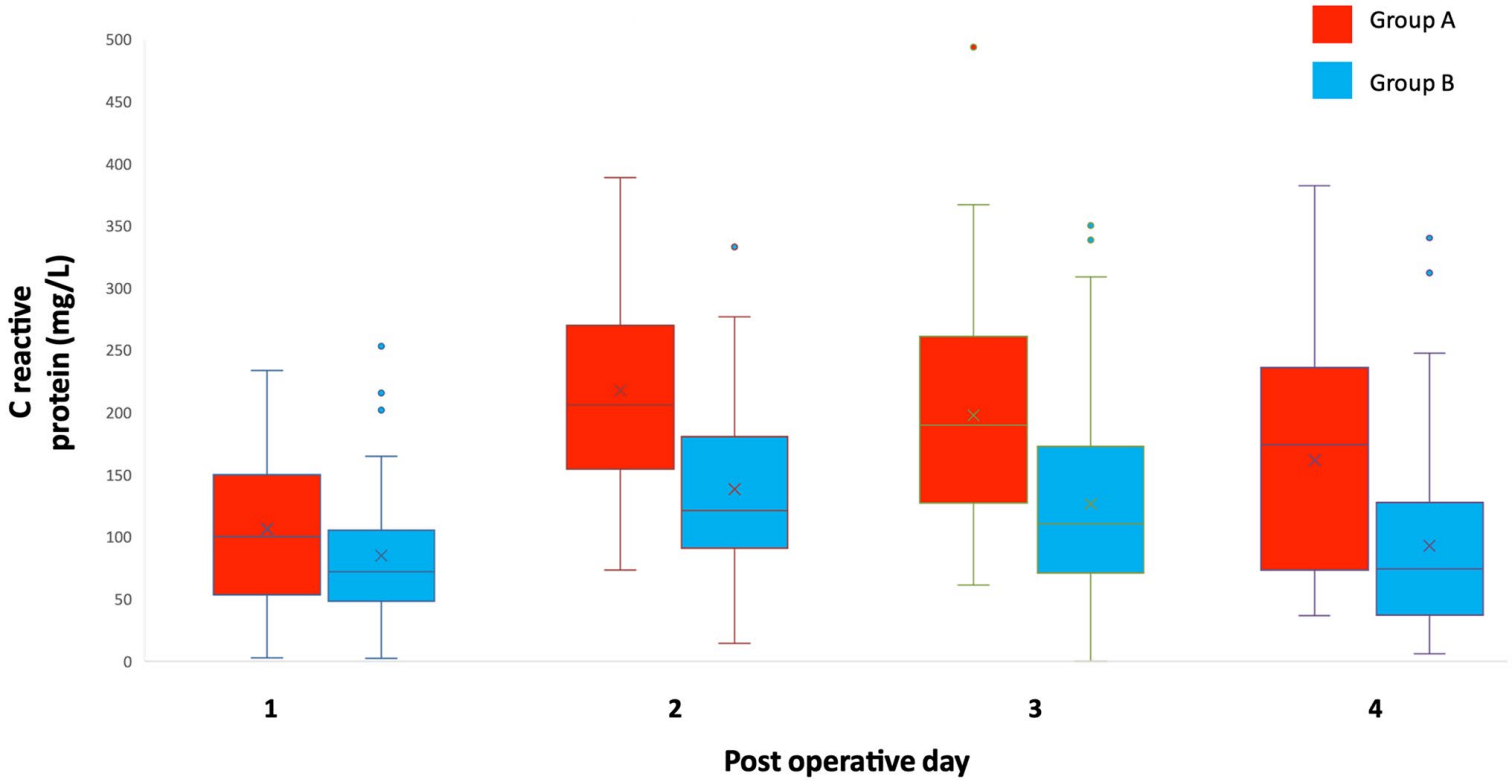
Comparison of C reactive protein between Group A (red) and Group B (blue) using box and whisker plot (Color figure online)

Patients in Group A (required longitudinal split of specimen) were not different in age, BMI or indications for surgery compared to Group B (those who did not require longitudinal split). Group A had a significantly longer mean operating time (254 vs. 220 min, *p* < 0.01). Mean length of stay was significantly longer in this group (6.6 vs. 5.3 days, *p* = 0.02), although complication rate and anastomotic leak rate were similar between the two groups (Table 4). Group A had significantly higher mean CRP on day two (218 vs. 139 mg/L, *p* ≤ 0.01), day three (198 vs. 130 mg/L, *p* < 0.01) and day four (162 vs. 94 mg/L, *p* < 0.01) as represented in Fig. 2. They also had significantly higher mean WCC on days two to four, which were 17–23% greater. One patient (2%) in Group A developed a port-site infection; another patient (2%) had post-operative fever of unknown source, compared to three (3%) in Group B but this was not significant (*p* = 0.86).

**Table 4:** Comparison between patients in Group A (required specimen splitting) and Group B (non-split)

Outcome	Group A	Group B	*p*-Value
Number (%)	57 [33]	114 [67]	–
Mean operating time (mins [SD])	254 [59]	220 [56]	< 0.01
Mean WCC (× 10^9^/L) [SD]			
Day 1	12.8 [4.9]	11.8 [3.2]	0.25
Day 2	12.3 [3.9]	10.0 [3.1]	< 0.01
Day 3	10.6 [3.5]	9.0 [3.3]	0.02
Day 4	9.5 [2.9]	8.0 [2.9]	0.03
Mean CRP (mg/L) [SD]			
Day 1	107 [65]	86 [53]	0.14
Day 2	218 [83]	139 [75]	< 0.01
Day 3	198 [101]	130 [76]	< 0.01
Day 4	162 [97]	94 [74]	< 0.01
Mean length of stay (days [SD])	6.6 [5.6]	5.3 [2.3]	0.02
Anastomotic leak (*n* [%])	1 [1.8]	2 [1.8]	0.53
Other complications ~ (*n* [%])	2 [3.5]	6 [5.3]	0.57

*Mins* minutes, *WCC* white cell count, *CRP* C reactive protein, *n* number, *SD* standard deviation, ~ Clavien-Dindo III–IV

Staple technique, emergency surgery or indication for surgery did not demonstrate any significant difference on perioperative outcomes, mean WCC or mean CRP.

## Discussion

Diverticular disease is a benign pathology which occurs in the majority of adults as they age, causing complications in roughly 20%. Episodes of stasis, bacterial proliferation, toxin and gas production and mucosal injury result in diverticulitis, peridiverticular abscesses or free perforation with peritonitis, or fistulae into surrounding organs. These processes manifest at surgery as phlegmonous, inflamed and fibrotic colon and mesentery with a predictably large diameter [15]. This prospective study is the largest series of NOSE colectomy including both elective and emergency diverticular surgery. As we have demonstrated, the techniques of mesocolic defatting and/or longitudinally splitting to facilitate bulky specimen extraction broaden inclusion to complex and obese patients who can benefit from NOSE and avoid transabdominal extraction (TAE). On-table colonoscopy prior to laparoscopy did not present any additional technical challenges nor were there any perforations. Laparoscopically selecting the descending colon transection site is paramount; as it is not always possible to be proximal to all diverticuli, healthy, supple bowel between diverticuli should be chosen. The overall length of stay, low complication rate and low anastomotic leak rate affirm the established feasibility and benefits of laparoscopic NOSE [7].

Avoiding TAE is associated with numerous positive benefits to patients: lower post-operative pain scores, lower opioid usage, fewer wound complications, quicker return of bowel function, lower long-term risk of incisional hernias and improved cosmesis [16–19]. Although operating time is longer in NOSE compared to TAE—and foreseeably so if additional debulking maneuverers are required—randomised trials have shown that overall procedure and hospital stay costs are significantly lower in NOSE cases, which offers healthcare systems an additional financial incentive to encourage uptake [16]. It is essential to address concerns regarding incontinence or poorer bowel function after transrectal NOSE which have also been raised, although these do not seem to be supported by convincing literature. The rectum and anus exhibit robust compliance, and studies have demonstrated no significant difference in patient-reported bowel function or Wexner score following NOSE colectomy [17, 18]. Our data also support the liberal use of NOSE in patients regardless of body mass index (BMI), which is not a consistent indication in the existing literature [11, 20]. The maximum BMI in the present study was 47.8 kg/m^2^. It is known that obese patients are at significantly greater risk of wound complications and incisional hernias (even if a lower risk extraction site such as a Pfannenstiel is used), and thus, NOSE offers an attractive alternative to avoid such complications in this cohort [21, 22].

It is interesting that mean length of stay was 1.3 days longer in patients who required specimen splitting. We propose this may be due to a combination of two reasons: either the small volume of microscopic contamination led to a slower recovery of bowel function and diet upgrade, or it reflects the fact that these patients had significant inflammatory pathology and therefore would have required a longer post-operative recovery period regardless. As Constantino et al. [18] have shown, peritoneal contamination occurs in virtually all patients undergoing diverticular resection regardless of extraction method. By comparing 20–30 mL samples of peritoneal fluid in 17 NOSE resections to 9 non-NOSE resections who were matched in age, sex and comorbidities, they found no significant difference in peritoneal contamination or overall complication rates. In fact, only one of these 26 patients had a negative peritoneal fluid sample. To mitigate luminal bacterial soiling, our routine practice involves pre-operative mechanical bowel preparation followed by colonoscopic lavage with dilute iodine [23].

We uniquely report the difference in white cell count and CRP between two subgroups (longitudinal specimen splitting versus non splitting), which is detectable from post-operative day two, in keeping with an inevitably greater bacterial load being transmitted into the peritoneal cavity when the colonic lumen is split. At a cellular level, peritoneal mesothelial cells demonstrate impressive adaptability to peritonitis; they effectively clear contaminants into the systemic circulation by promoting margination and migration of neutrophils, and production of proinflammatory cytokines such as IL-6, IL-8 and TNF-α. Gaps within mesothelial cells double or triple in size to facilitate clearance of contaminated peritoneal fluid into lymphatic lacunae [24]. Although this process begins within minutes, our study is the first to objectively demonstrate that even modest peritoneal contamination from an open (or perforated) colon may only be detectable on systemic pathology tests after 24–48 h, since day one results were not significantly different between subgroups. Elevated levels of CRP in NOSE patients does not appear to correlate to infective complications and must be interpreted in the context of clinical findings. For example, CRP trajectory is a more sensitive marker than isolated values for anastomotic leakage [25, 26].

This study is strengthened by the prospective design and large sample size. Existing studies exclusively evaluating outcomes for diverticular resections have included sample sizes of less than 30 patients [18, 27, 28]. We acknowledge it is limited by the absence of a comparator group who underwent TAE, which would allow direct comparison to traditional specimen retrieval. The addition of clinical outcomes to reflect bowel function and pain scores may provide further insights on the impact of peritoneal inflammation. The implication for future research is to encourage a shift in laparoscopic NOSE colectomy with intracorporeal anastomosis from a ‘novel’ procedure to a technically achievable alternative for complex benign or malignant indications, with or without a robotic platform.

NOSE colectomy for diverticular disease has excellent perioperative outcomes in this large series. Specimen debulking is commonly required due to the inflammatory nature of this condition, which can allow more patients to successfully undergo NOSE colectomy and achieve the associated benefits. If specimen splitting is required, higher white cell count and C reactive protein levels are expected post-operatively but do not correlate to an increased complication rate.

## Data Availability

Dataset generated in this study is available upon reasonable written request to the corresponding author.
